# Proposal of a rhombohedral-tetragonal phase composition for maximizing piezoelectricity of (K,Na)NbO_3_ ceramics

**DOI:** 10.1038/s41598-019-40943-6

**Published:** 2019-03-12

**Authors:** Min-Ku Lee, Sun-A Yang, Jin-Ju Park, Gyoung-Ja Lee

**Affiliations:** Nuclear Materials Development Division, Korea Atomic Energy Research Institute, Daejeon, 34057 Republic of Korea

## Abstract

Great progress in the field of piezoelectricity of (K,Na)NbO_3_ (KNN) lead-free ceramics, driven by emerging rhombohedral-tetragonal (R-T) phase boundary, has instigated research activity regarding elaborate controls of the phase boundary structure. Through phase-microstructure-property mapping in KNN ceramics doped with Bi-containing perovskite oxides, in this study we for the first time report the existence of a certain R-T phase boundary state by which to create maximum piezoelectric response in KNN systems. This phase boundary condition is usually comprised of approximately 15% R phase and 85% T phase, regardless of the choice of dopant material. Any deviation from this phase composition, either by inclusion of orthorhombic (O) phase or by enrichment of R phase, has a negative effect on the value of *d*_33_. These findings can provide useful guidance for chemical doping control associated with the type of phase boundary and the phase composition for advanced KNN-based materials.

## Introduction

A great deal of effort is being expended to develop viable and competitive lead-free piezoelectric materials. Among potential materials, KNN-based ceramics are widely considered a great candidate owing to excellent piezoelectric coefficient *d*_33_ and high Curie temperature *T*_C_^1–3^. Benefitting from facilitated polarization rotations for a state in which plural structural phases coexist with negligible difference in free energy^4^, research concerning the construction of a phase boundary at/near room temperature has been extensively pursued during the last decade by means of chemical modification.

It is well accepted that the shift of two intrinsic polymorphic phase transitions of KNN, i.e., the rhombohedral (R)-orthorhombic (O) transition at −123 °C (*T*_R-O_) and the orthorhombic (O)-tetragonal (T) transition at 200 °C (*T*_O-T_), close to room temperature can be possible when KNN materials are doped with special ions (Li^+^, Sb^5+^ and Ta^5+^) or/and ABO_3_-type perovskite compounds with different valence states of A- and B-sites, leading to enhanced piezoelectric activity owing to the formation of a phase boundary at room temperature. Because of great improvements of *d*_33_ to more than 300 pC/N, keen attention has been paid to the construction of the O-T phase boundary, rather than to the R-O phase boundary^5–9^.

The surprisingly high room-temperature *d*_33_ values (360–570 pC/N) recently achieved by the novel R-T^10–16^ and R-O-T phase boundaries^17–20^ have triggered strong interest in developing a new KNN system. To create such phase boundaries, more complicated compositional manipulation by means of two or more additives is typically involved, such that the discrete *T*_R-O_ and *T*_O-T_ values concurrently move to room temperature. Not only the proper choice of dopant material but also the meticulous control of material compositions is found to be critical to constructing the phase boundary. The highest *d*_33_ value (570 pC/N) reported to date among the randomly-oriented KNN-based ceramics is achieved by constructing a R-T phase boundary by doping with Sb^5+^, BaZrO_3_ and (Bi,K)HfO_3_^10^. Nevertheless, this marked progress in *d*_33_ is achieved at the expense of *T*_C_ (which has a value less than 200 °C), similar to the case of conventional lead zirconium titanate [Pb(Zr,Ti)O_3_ (PZT)] ceramics.

Even though the current upsurge of research interest has focused on these emerging phase boundaries, little is known about the exact role of ferroelectric R, O, and T phases or their coexistence constructing a phase boundary in piezoelectricity. This is mainly due to the lack of evidence for an actual phase and its quantitative composition in the involved phase boundaries, as most structural considerations for phase boundaries have been made by qualitative methods of research, typically based on XRD peak analysis coupled with analysis of temperature-dependent dielectric constant behaviors.

In this study, we fully illuminate the fundamental roles of ferroelectric R, O, and T phases and their coexistence in the piezoelectric activity and Curie temperature (*T*_C_) of KNN-based ceramics. On this basis, a certain R-T phase boundary condition to maximize the piezoelectric response is ultimately proposed. With regard to the control of the phase boundary structure, a comprehensive chemical modification of KNN is conducted by doping Bi-containing perovskite oxides (Bi*M*O_3_, *M* = A- or/and B-site elements); this modification allows us to prepare ferroelectric R, O, and T phases constructing a phase boundary in different ratios. Perovskite oxides including Bi^3+^ ion are of special interest in lead-free piezo-/ferroelectrics and multiferroic materials^21^, because the Bi^3+^ ion has a stereochemically active 6 s^2^ lone pair, which results in structural distortion of the prototypical cubic phase owing to ion-off centering in its perovskite compounds, as is the case for the Pb^2+^ ion. Accordingly, these materials are currently in general use as doping agents for KNN and, besides, the doping with these compounds as end members has been shown to be very useful to tune the intrinsic phase transition temperatures of KNN^2,3^. Among Bi*M*O_3_ compounds, in this work we introduce Bi_0.5_(Li_0.7_Na_0.2_K_0.1_)_0.5_ZrO_3_ (BNKLZ), in which dopants of Bi^3+^ and Zr^4+^ ions are known to play effective roles in decreasing *T*_O-T_ and increasing *T*_R-O_, respectively^22,23^. Additionally, perovskite oxides of BiScO_3_ (BS), (Bi_0.5_Na_0.5_)TiO_3_ (BNT), and BiGaO_3_ (BG) are doped for further control of the phase boundary structure. The constituent phases and their actual compositions in the induced phase boundaries are thoroughly evaluated via the Rietveld refinement method.

## Results and Discussion

**Figure 1:**
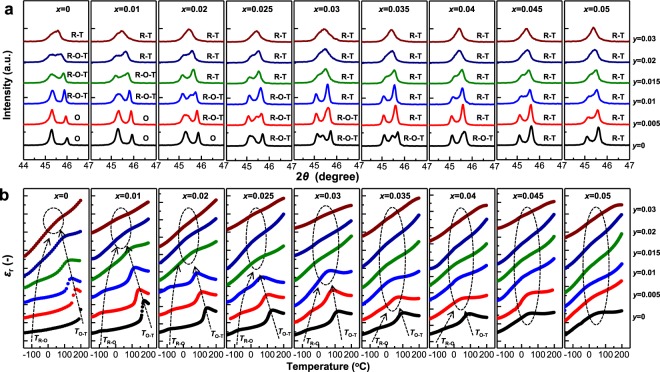
(**a**) Amplified XRD patterns of (1 − *x* − *y*)KNN-*x*BNKLZ-*y*BS ternary ceramics (*x* = 0–0.05, *y* = 0–0.03) in the 2*θ* range of 44–47° measured at room temperature. The results of phase structure as identified by Rietveld refinements are presented in (**a**). (**b**) Temperature dependence of dielectric constant *ε*_r_ measured at 100 kHz in the temperature range of −150 to 200 °C. The dotted circles indicate regions corresponding to coexistence of R and T phases. The *T*_R-O_ and *T*_O-T_ converge at/near room temperature by doping with BNKLZ (*x*) and BS (*y*).

First, based on the room-temperature XRD patterns, it was confirmed that the (1 − *x* − *y*)KNN-*x*BNKLZ-*y*BS ternary ceramics (*x* = 0–0.05, *y* = 0–0.03) were a pure perovskite structure without second phases (Fig. S1). As a result, both the BNKLZ and BS dopants completely diffused into the KNN lattice, forming a stable solid solution in the investigated *x* and *y* ranges. To identify the structural evolution with the variation of *x* and *y*, Rietveld analysis was carried out for the corresponding XRD patterns. The analyzed phases are presented along with the amplified (2*θ* = 44–47°) XRD patterns (Fig. 1a). For ceramics with low *x* (0 ≤ *x* ≤ 0.02), the O phase initially appears and transforms via a triphasic R-O-T structure into a diphasic R-T structure, as *y* increases. For ceramics with 0.025 ≤ *x* ≤ 0.04, the transition of R-O-T into R-T occurs with an increase in *y*, accompanied by the shrinkage of the O phase. At higher *x* values (*x* ≥ 0.045), the O phase completely disappears, leaving only the coexisting R and T phases. In the R-T regions, gradual peak merging between tetragonal 002 and 200 reflections with increasing *x* and *y* values is likely induced by the increased content of R phase relative to T phase.

Phase transition temperatures and phase structures for ceramics with different *x* and *y* values were characterized by temperature-dependent dielectric constant (*ε*_r_ − *T*) measurement from −150 to 200 °C (Fig. 1b). Two dielectric anomalies involving the phase transitions of R-O and O-T are clearly separated in the absence of BNKLZ and BS. The measured *T*_R-O_ and *T*_O-T_ values are −120 and 200 °C, respectively, similar to the values in the literature for KNN; the O phase forms at room temperature. Both the transition peaks gradually approach the room-temperature region as the *x* and *y* values increase. Finally, the peaks converge to create a single and broad maximum peak centered at/near room temperature. The appearance of a single dielectric peak at room temperature indicates that, owing to the extinction of O phase, only the R and T phases coexist, eventually constructing the R-T phase boundary.

The critical dopant concentrations required to form a single dielectric peak at room temperature are as follows: *x* = 0 and *y* = 0.03, *x* = 0.01 and *y* = 0.02, *x* = 0.02 and *y* = 0.015, *x* = 0.03 and *y* = 0.01, *x* = 0.04 and *y* = 0.005, and *x* = 0.05 and *y* = 0. These critical *x* and *y* values are consistent with those corresponding to the formation of a pure R-T structure in the above XRD patterns (Fig. 1a). The decreased critical *y* values with increasing *x* (and *vice versa*) indicate that there are suitable doping levels (0.03–0.05 based on the sum of *x* and *y* values) to produce an R-T structure with the removal of the O phase. It can also be seen that, compared to doping of BNKLZ, the doping of BS results in more rapid change in the phase structure. Considering the elemental influence of the Bi*M*O_3_ perovskites, this difference is believed to be due to the effect of different concentrations of Bi^3+^ ion between the BS and BNKLZ compounds, hinting at a strong contribution of Bi^3+^ to the formation of the phase boundary structure. The mole fractions of Bi^3+^ ion required for the coexistence of R and T phases are between 0.025–0.03. Further increase in *x* and *y* above these values can induce dielectric peak suppression, which can be correlated with XRD peak merging owing to increased R phase content (Fig. 1a). All the *ε*_r_ − *T* curves strongly support the structural evolution observed in the XRD patterns. Consequently, both the XRD and *ε*_r_ − *T* measurements confirm that the doping of BNKLZ and BS effectively promotes the shrinkage of the O phase, ultimately creating the R-T phase boundary near room temperature via the formation of a R-O-T structure. Similar phase evolution (O → R-O-T → R-T → R-rich) induced by chemical doping is observed in KNN ceramics doped with BNKZ and Sb^11^.

**Figure 2:**
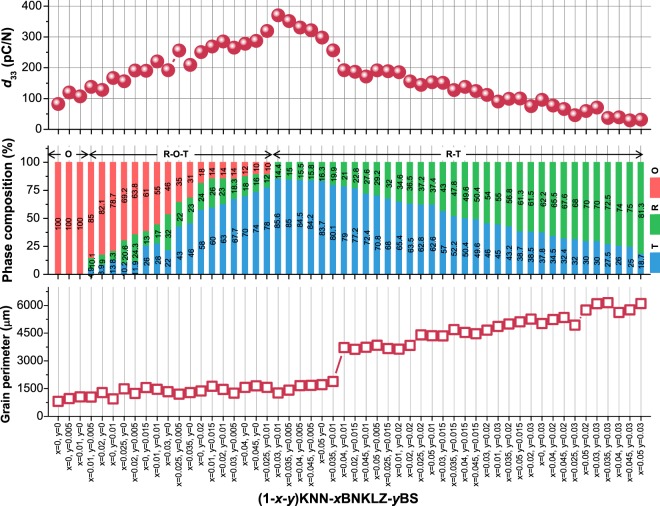
Correlation of piezoelectric coefficient *d*_33_ with phase composition and grain perimeter length results for (1 − *x* − *y*)KNN-*x*BNKLZ-*y*BS ternary ceramics (*x* = 0–0.05, *y* = 0–0.03). The quantitative composition data (R, O, and T phases) identified by Rietveld refinements are given in this figure.

To manifest the role of each ferroelectric phase (R, O, and T phases) of a phase boundary in the piezoelectric performance, Rietveld refinement analysis is used to determine the quantitative compositions of constituent phases for the ceramics prepared as a function of the BNKLZ (*x*) and BS (*y*) contents (Fig. S2 and Table S1). Together with room-temperature measurements of *d*_33_, the values of grain perimeter length (or grain boundary length) are also estimated for the corresponding ceramics (Fig. S3). This enables mapping of the phase boundary structure (constituent phases and their composition), grain perimeter length (grain size) and piezoelectric *d*_33_ properties of the KNN-*x*BNKLZ-*y*BS ternary ceramics. Finally, a cross-correlation analysis of such maps establishes a correlation shown in Fig. 2.

The most significant finding from this correlation is that the involved phases and their compositions impact greatly on the *d*_33_ property. Given the well-established grain-size effect in lead-based piezoceramics, it should be noted that the *d*_33_ behavior does not tally with the grain size behavior: note that the variation of grain perimeter length is relatively small in the O and R-O-T regions, whereas the *d*_33_ trend shows a clear increase in the same structural regions. Meanwhile, the observed *d*_33_ behavior is strongly influenced by the nature of the phase and its quantitative composition. Overall, the *d*_33_ value increases with decreases in content of O and R phases, accompanied by simultaneous increase of the T phase. More importantly, the *d*_33_ value reaches its maximum (370.2 pC/N), when the phase boundary possesses 14.4% of R phase and 85.6% of T phase, without O phase.

The *d*_33_ behavior, directly affected by the phase structure, can be clearly seen in Fig. S4 (redrawn from Fig. 2), showing the concentration dependency of the dopants. The peaking behavior of *d*_33_ is evident; it is induced by the decrease in the content of O and R phases and the simultaneous increase in the T phase. To obtain a higher *d*_33_ value at the peak, Fig. S4 also implies that it is particularly important for a phase boundary to be controlled so as to have a phase composition close to 15% R phase and 85% T phase. This R-T phase boundary condition is not easily obtained, because the types and compositional progress of formed phases are sensitive to the dopant material and its concentration. Indeed, an optimal R-T phase composition (15% R phase-85% T phase) appears almost immediately after the O phase disappears during R-O-T phase coexistence, showing the necessity of precise doping control in the vicinity of the transition zone from R-O-T to R-T structure. In this work, only a few combinations of *x* and *y* values can lead to this phase boundary condition, with the highest *d*_33_ values between 320–370 pC/N. Namely, these values are *x* = 0.03 and *y* = 0.01, *x* = 0.035 and *y* = 0.005, *x* = 0.04 and *y* = 0.005, and *x* = 0.045 and *y* = 0.005 (0.04–0.05 based on total mole fractions of dopants). Consequently, the results shown in Figs 2 and S4 suggest the existence of a particular phase boundary condition that can lead to enhanced piezoelectric activity of KNN ceramics.

On the other hand, one can see in Fig. 2 that the grain perimeter length increases suddenly for the ceramic with *x* = 0.04 and *y* = 0.01. As has been shown for many KNN systems^24–26^, low levels of doping can promote grain growth via the formation of a liquid phase caused by low-melting-point Bi; however, at high doping levels the dopants can locate at the grain boundaries and, owing to their solubility limit, prohibit grain growth. Accordingly, the corresponding ceramic exhibits a sharp decrease in *d*_33_ because the increased grain boundaries generally constrain the domain wall motion. In terms of phase composition, the observed grain size reduction arises when the R phase content exceeds about 20%; this is followed by progressive reduction in the grain size with further increase in the R phase content. In addition to the grain size reduction, the increased R phase content is another contribution to the decline of *d*_33_ observed in the R-T region. This result directly shows the need for the determination of suitable doping levels to produce the optimum R-T phase composition. Otherwise, the phase boundary becomes enriched above the optimum level with R phase, accompanied by grain size reduction.

To confirm the existence of the optimum phase boundary in other doped KNN systems, different dopant materials of BNT and BG were added instead of BS to the KNN-BNKLZ system. Similar to the KNN-BNKLZ-BS system, both the KNN-*x*BNKLZ-*y*BNT and KNN-*x*BNKLZ-*y*BG systems showed peaking behavior of *d*_33_ induced by decrease in the content of O and R phases (Figs S5a,b). The measured *d*_33_ peak values are lower (260.1 pC/N for BNT doping and 302.7 pC/N for BG doping) than that obtained by BS doping. However, the highest piezoelectric response is likewise acquired from similar R-T phase compositions: 13.2% R phase and 86.8% T phase for BNT doping and 14% R phase and 86% T phase for BG doping. The corresponding mole fractions of the dopants (sum of the *x* and *y* values) are 0.045 and 0.047 for the BNT and BG dopings, respectively; these values are similar to those for BS doping. From the results shown in Figs 2 and S5, it is reasonable to assume that the R-T phase composition guaranteeing a maximum piezoelectric response is almost constant independent of the dopant material, although the obtainable *d*_33_ peak value may change depending on the dopant material. Furthermore, as observed for the KNN-0.03BNKLZ-0.02BNT ceramic (Fig. S6a), increased T phase content up to 93.3% in the R-T phase boundary at room temperature, owing to the drop of *T*_R-T_ (i.e., rhombohedral-tetragonal transition) below room temperature, results in decreased *d*_33_ (Fig. S5a). This can be complementary evidence for the existence of the aforementioned optimum R-T phase composition, which might have been missed in previous studies on chemically doped KNN systems.

For KNN-based ceramics, piezoelectric sensitivity of a phase composition in coexisting phases has rarely been reported on, excluding a few studies. Ahn *et al*. first stressed the need to construct a T-rich O-T phase boundary for piezoelectric enhancement, although their structural evidence was inconclusive given the phase composition determination^27^. As for the R-T phase boundary, Lv *et al*. recently reported that a higher T phase content (up to about 80%) benefits the piezoelectric performance, which is induced by larger degrees of lattice distortion and induced internal stress produced by T phase rather than by R phase^28^. According to recent *in-situ* synchrotron XRD work, there is a close relationship between an electric-induced phase transition and the piezoelectricity of KNN ceramics^13,29–31^; the enhanced piezoelectricity achieved by a T-rich R-T phase boundary is attributed to the more positive role of the irreversible tetragonal-electric induced phase transition compared to the rhombohedral-electric induced phase transition with a certain degree of reversibility^13^. It has also been confirmed via Rayleigh analysis (Fig. S7a–c) that the intrinsic (lattice distortion or reversible domain wall vibration) and extrinsic (irreversible motion of domain wall or phase boundaries) contributions to piezoelectric activity are highest at optimum R-T phase composition. Our findings are reasonably in line with the previous investigations, providing further conclusive evidence for an ideal phase composition of the R-T phase boundary. Despite our efforts to elucidate the roles of the phase boundary structure, the physical mechanism of such a particular T-rich phase composition, related to the piezoelectric enhancement, will require further investigation.

**Figure 3:**
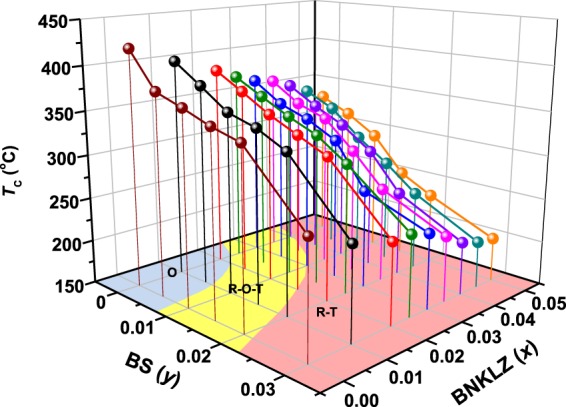
Variation of Curie temperature *T*_C_ as function of *x* and *y* values for (1 − *x* − *y*)KNN-*x*BNKLZ-*y*BS ternary ceramics (*x* = 0–0.05, *y* = 0–0.03).

In Fig. 3, the values of Curie temperature (*T*_C_) determined from the dielectric peak positions (Fig. S8) are plotted as a function of both the *x* and *y*. A gradually decreasing trend in *T*_C_ can be seen with the increase of the *x* and *y* values, indicating that the addition of BNKLZ and BS lowers *T*_C_. With increase of *x* and *y* up to 0.05 and 0.03, respectively, the *T*_C_ value drops from 420 °C to 200 °C. Regarding the effects of the dopant, the decrease rate of *T*_C_ is found to be higher for BS doping (34.7 ± 2.0 °C/0.01 mol) than for BNKLZ doping (15.8 ± 2.3 °C/0.01 mol). In the cases of BNT and BG doping, the decrease rates of *T*_C_ are estimated to be 15.0 ± 1.7 °C/0.01 mol and 69.9 ± 7.4 °C/0.01 mol, respectively (data not shown here). As a result, the choice of dopant material can have a critical impact on the decrease rate of *T*_C_ (BG > BS > BNKLZ ≈ BNT). It is also revealed that the dopant materials (e.g. BNKLZ and BNT), which are structurally similar to and have the same chemical valences of A- and B-sites, can have similar effects on *T*_C_, as compared to BS and BG. The decrease rates of *T*_C_ observed for BNT or BNKLZ doping in this work are nearly comparable to those reported by previous studies^19,32,33^.

It is further noted that the *T*_C_ values for ceramics possessing near-optimum phase compositions (around 15% R and 85% T) remain almost unchanged with Δ*T*_C_ ≈ 9 °C: ~332 °C for *x* = 0.03 and *y* = 0.01, ~335 °C for *x* = 0.035 and *y* = 0.005, ~331 °C for *x* = 0.04 and *y* = 0.005, and ~326 °C for *x* = 0.045 and *y* = 0.005. Despite differences of microstructure, grain size, and piezoelectric/ferroelectric properties among those samples, the observed steady *T*_C_ characteristic can be attributed to structural similarities in phase structure, phase composition, tetragonality, and so on (Fig. S9). The present levels of *d*_33_ (~370 pC/N) and *T*_C_ (~332 °C) values achieved by constructing the optimum R-T phase boundary condition are not only almost record-high compared with those of the recently developed polycrystalline KNN ceramics, which have high *T*_C_ values above 300 °C (Fig. S10)^2,3^, but are also comparable to those of commercial ‘soft’ PZT ceramics^34^.

Even if the ceramics are controlled to have a near-optimum R-T phase composition, the maximum obtainable *d*_33_ value can be different (260–370 pC/N) depending on the applied dopant material as mentioned earlier. On the contrary, *T*_C_ values for ceramics with near-optimum R-T phase composition produced at relatively modest doping levels of Bi*M*O_3_ (0.04–0.05 in the mole fraction) not only remain at high levels of 332–343 °C, but are also almost unchanged for the three KNN ternary ceramic systems (Fig. S11). In consideration of the universal trade-off relationship between the two technologically important properties, *d*_33_ and *T*_C_, it is therefore promising that achieving an optimum R-T phase boundary structure by way of doping with Bi-containing perovskites (Bi*M*O_3_) can benefit the balanced development of *d*_33_ and *T*_C_ in KNN-based ceramics. With the great emphasis on the importance of the manipulation of the phase composition, the observed piezoelectric sensitivity to different dopant materials certainly encourages us to work concerning the construction of an optimum R-T phase boundary by employing other beneficial dopant materials.

Through phase-microstructure-property mapping in KNN piezoceramics doped with various useful Bi-containing perovskite oxides (Bi*M*O_3_), this work proposes some novel ideas on doping control for developing new KNN-based ceramics as follows:

1. For doping-induced structural progress (O → R-O-T → R-T → R-rich), it is particularly important for the phase boundary structure to be optimized to have a particular phase composition of about 15% R and 85% T to maximize the piezoelectric response. As obtained at modest doping levels, this optimum R-T phase composition can also guarantee high *T*_C_ values without serious doping-induced loss, enabling balanced development between the *d*_33_ and *T*_C_ properties (*d*_33_ ~370 pC/N, *T*_C_ ~332 °C in this work).
2. To obtain the optimum R-T phase composition (15% R-85% T), precise doping control is necessary in the vicinity of the transition zone from the R-O-T to R-T structure because this optimum condition usually arises immediately after the O phase disappears from the R-O-T structure during structural progress. Particular attention should be paid to doping control for dopant materials whose inputs have a sensitive influence on the type and compositional progress of the forming phases (for instance, BiGaO_3_).
3. When the phase boundary structure of an existing material system is R-O-T rather than R-T, there is more room to use additional dopant materials to improve a *d*_33_ peak value. When the ceramics possess an R-T phase boundary with more than 15% of R, however, doping control is no longer effective because further doping will certainly induce a R-richer phase boundary, accompanied by decreased grain sizes, with poor piezoelectric performance.

## Methods

### Materials

(1 − *x* − *y*)KNN-*x*BNKLZ-*y*BS (*x* = 0–0.05, *y* = 0–0.03) ternary ceramics including (1 − *x* − *y*) KNN-*x*BNKLZ-*y*BNT (*x* = 0.03, *y* = 0–0.04) and (1 − *x* − *y*)KNN-*x*BNKLZ-*y*BG (*x* = 0.045, *y* = 0–0.01) were prepared using the conventional solid-state powder method. The starting powders were K_2_CO_3_ (≥99.0%, 150 μm, Sigma-Aldrich), Na_2_CO_3_ (≥99.5%, 10 μm, Sigma-Aldrich), Li_2_CO_3_ (99.997%, 20 μm, Sigma-Aldrich), Nb_2_O_5_ (99.9%, 2 μm, Sigma-Aldrich), Bi_2_O_3_ (99.9%, 10 μm, Sigma-Aldrich), ZrO_2_ (99.0%, 5 μm, Sigma-Aldrich), Sc_2_O_3_ (≥99.9%, 10 μm, Sigma-Aldrich). TiO_2_ (≥99.9%, 5 μm, Sigma-Aldrich) and Ga_2_O_3_ (≥99.99%, 10 μm, Sigma-Aldrich). With an eye to practical applications, toxic Sb and high-cost Ta, although in common use and useful as doping elements for KNN, were avoided in this work.

### Synthetic procedures

Stoichiometric powder mixtures with different *x* and *y* concentrations were homogenized in ethanol for 24 h using a roller mill machine operating at 70 rpm and dried at 120 °C for 24 h. All treatments of these powders, including weighing and drying, were carefully performed within a glove box filled with Ar gas. Calcination was then carried out at 850 °C for 6 h. The calcined powders were mixed with poly-vinyl alcohol (PVA) as a binder and axially compacted into disks 10 mm in diameter. After burning off the PVA at 650 °C, traditional pressureless sintering was carried out at 1100 °C for 3 h in air. The sintered ceramics were ground to a thickness of 1 mm. The resulting ceramics had similar densities of 4.25–4.32 g/cm^3^ (greater than 95% of the theoretical density), determined based on the Archimedes method.

### Phase structure

The crystal structures of the unpoled samples were characterized by an X-ray diffractometer (XRD; D/Max-2500; Rigaku, Tokyo, Japan) using Cu *K*α radiation at a power of 40 kV and 15 mA and at a scan speed of 1°/min. Rietveld refinement of XRD patterns was performed to accurately determine the phases and their quantitative compositions using Rigaku’s PDXL software with the Whole Pattern Powder Fitting (WPPF) method, connected to Inorganic Crystal Structure Database (ICSD; FIZ Karlsruhe, Germany). The initial models used for refinement were three KNbO_3_ crystal structures, i.e., orthorhombic (*Amm*2; JCPDS 01–071–0946; ICSD code 9533), rhombohedral (*R*3*m*; JCPDS 01–071–0947; ICSD code 9534), and tetragonal (*P*4*mm*; JCPDS 01–071–0948; ICSD code 9535); their crystal structure parameters were acquired from the ICSD. The refinements were carried out by checking the fitting quality of the powder patterns by means of two parameters, i.e., the goodness-of-fit indicator *S* and the reliability factor *R*_wp_. Here, the low *R*_wp_ of less than 10% as well as the *S* value close to 1 ensure the high reliability of the refinement results.

### Grain structure

The morphology of the sintered samples was studied using a field-emission scanning electron microscope (FE SEM; Sirion; FEI, Eindhoven, Netherlands) with an operating voltage of 20 kV equipped with an energy-dispersive X-ray spectrometer (EDXS). Since the grain boundaries generally play a role of hindering the domain wall motion, the values of grain perimeter length (or grain boundary length), together with the grain size, were estimated to investigate the effect of the grain boundary area on the piezoelectric response for all sintered ceramics. The quantitative measurements were carried out using automatic image analysis software (Matrox imaging library 10) for SEM images taken from each sample: number of analyzed images = 4–8 per sample, measurement area per image = 19.1 × 25.6 μm, total number of grains = about 400 to several thousands.

### Electrical characterization

For the electrical measurements of the sintered ceramics, both sides of the disks were polished and painted with a silver paste, after which disks were fired at 650 °C for 10 min. The samples were then poled in silicon oil at room temperature. After optimization through pre-tests of poling over time and in an electric field, poling field and time were set at 40 kV/mm and 10 min, respectively. Dielectric constant *ε*_r_ at low temperatures ranging from −150 to 200 °C was measured for the unpoled samples using an impedance analyzer (HP 4294 A; Agilent; USA) at a frequency of 100 kHz. Measurements between 30–500 °C were also conducted using an impedance analyzer (SI 1260; Solartron; Farnborough, England) combined with a dielectric interface (SI 1287; Solartron; Farnborough, England). Hence, the effects of the addition of Bi*M*O_3_ dopants on the phase transition temperatures (*T*_R-O_, *T*_O-T_ and *T*_C_) of KNN were fully characterized. The longitudinal piezoelectric coefficients (*d*_33_) were measured for the poled samples at room temperature using a piezo-*d*_33_ meter (ZJ-6B; IACAS; Beijing, China).

## Supplementary information

supplementary information
