# Supplementary material for: Proposal of a rhombohedral-tetragonal phase composition for maximizing piezoelectricity of (K,Na)NbO3 ceramics
Source: Sci Rep. 2019 Mar 12;9:4195. doi: 10.1038/s41598-019-40943-6 (PMC6414638; doi:10.1038/s41598-019-40943-6)
Supplement: Supplementary file 1 — supplementary information [file 41598_2019_40943_MOESM1_ESM.doc]

**Supplementary Information**

Proposal of a rhombohedral-tetragonal phase composition for maximizing piezoelectricity of (K,Na)NbO_3_ ceramics

Min-Ku Lee, Sun-A Yang, Jin-Ju Park, & Gyoung-Ja Lee^*^

Nuclear Materials Development Division, Korea Atomic Energy Research Institute, Daejeon 34057, Republic of Korea

* corresponding author: [leegj@kaeri.re.kr](mailto:leegj@kaeri.re.kr)

**Figure S1.** Room-temperature XRD patterns of the (1 – *x* – *y*)KNN-*x*BNKLZ-*y*BS ternary ceramics in the 2*θ* range of 20 – 60°. a) *x* = 0 – 0.05, *y* = 0. b) *x* = 0.03, *y* = 0 – 0.03.

The normal *θ*-2*θ* XRD scans were carried out at room temperature for all the (1 – *x* – *y*)KNN-*x*BNKLZ-*y*BS ternary ceramics (*x* = 0 – 0.05, *y* = 0 – 0.03). All the sintered ceramics possessed a pure perovskite structure without second phases, confirming the formation of a complete solid solution among KNN, BNKLZ and BS in the investigated *x* and *y* ranges. Figures S1a and b are the typical XRD patterns (2*θ* = 20 – 60°) showing the formation of a pure perovskite structure.

| Sample | *x* = 0.03, *y* = 0.01 |
| --- | --- |
| Space group | *R*3*m* |
|  | *P*4*mm* |
| Cell parameter for *R*3*m* (Å) | 3.9786 |
|  | 3.9786 |
|  | 3.9786 |
| Cell parameter for *P*4*mm* (Å) | 3.9717 |
|  | 3.9717 |
|  | 4.0133 |
| Tetragonal phase (%) | 85.6 |
| Rhombohedral phase (%) | 14.4 |
| *R*_wp_ (%) | 8.83 |
| *S* | 1.4171 |

**Figure S2.** Rietveld refinement of the XRD pattern of the (1 – *x* – *y*)KNN-*x*BNKLZ-*y*BS ceramic with *x* = 0.03 and *y* = 0.01. The results of cell parameters, phase compositions and reliability factors, *R*_wp_ and *S*, are listed in the table below.

**Table S1.** Quantitative phase compostitions and reliability factors obtained from the Riedveld refinements of the the (1 – *x* – *y*)KNN-*x*BNKLZ-*y*BS ternary ceramics (*x* = 0.01 – 0.05, *y* = 0 – 0.03). The R, O, and T indicate the phase contents of rhombohedral, orthorhombic, and tetragonal phases.

| *x*=0 | *R*_wp_ (%) | *S* | R | O | T |  | *x*=0.01 | *R*_wp_ (%) | *S* | R | O | T |
| --- | --- | --- | --- | --- | --- | --- | --- | --- | --- | --- | --- | --- |
| *y*=0 | 7.88 | 1.2619 | - | 100 | - |  | *y*=0 | 9.42 | 1.5389 | - | 100 | - |
| *y*=0.005 | 8.50 | 1.3590 | - | 100 | - |  | *y*=0.005 | 8.44 | 1.3382 | 10.1 | 85.0 | 4.9 |
| *y*=0.01 | 8.28 | 1.3195 | 8.3 | 78.7 | 13.0 |  | *y*=0.01 | 9.81 | 1.5740 | 17.0 | 55.0 | 28.0 |
| *y*=0.015 | 8.44 | 1.3382 | 13.0 | 61.0 | 26.0 |  | *y*=0.015 | 8.13 | 1.2809 | 26.0 | 14.0 | 60.0 |
| *y*=0.02 | 7.22 | 1.1346 | 24.0 | 18.0 | 58.0 |  | *y*=0.02 | 7.71 | 1.2175 | 34.6 | - | 65.4 |
| *y*=0.03 | 7.0 | 1.0933 | 37.8 | - | 62.2 |  | *y*=0.03 | 7.26 | 1.1402 | 55.0 | - | 45.0 |

| *x*=0.02 | *R*_wp_ (%) | *S* | R | O | T |  | *x*=0.025 | *R*_wp_ (%) | *S* | R | O | T |
| --- | --- | --- | --- | --- | --- | --- | --- | --- | --- | --- | --- | --- |
| *y*=0 | 8.71 | 1.4157 | 9.0 | 82.1 | 8.9 |  | *y*=0 | 9.71 | 1.5561 | 20.6 | 69.2 | 10.2 |
| *y*=0.005 | 11.5 | 1.8486 | 24.3 | 63.8 | 11.9 |  | *y*=0.005 | 9.80 | 1.5728 | 22.0 | 35.0 | 43.0 |
| *y*=0.01 | 8.91 | 1.4238 | 23.0 | 14.0 | 63.0 |  | *y*=0.01 | 9.58 | 1.5216 | 12.0 | 10.0 | 78.0 |
| *y*=0.015 | 7.49 | 1.1874 | 22.8 | - | 77.2 |  | *y*=0.015 | 9.63 | 1.5180 | 32.0 | - | 68.0 |
| *y*=0.02 | 6.39 | 1.0094 | 36.5 | - | 63.5 |  | *y*=0.02 | 8.68 | 1.3636 | 37.2 | - | 62.8 |
| *y*=0.03 | 6.66 | 1.0476 | 61.5 | - | 38.5 |  | *y*=0.03 | 8.98 | 1.4072 | 68.0 | - | 32.0 |

| *x*=0.03 | *R*_wp_ (%) | *S* | R | O | T |  | *x*=0.035 | *R*_wp_ (%) | *S* | R | O | T |
| --- | --- | --- | --- | --- | --- | --- | --- | --- | --- | --- | --- | --- |
| *y*=0 | 9.56 | 1.5391 | 32.0 | 46.0 | 22.0 |  | *y*=0 | 9.84 | 1.5915 | 23.0 | 31.0 | 46.0 |
| *y*=0.005 | 8.95 | 1.4285 | 18.3 | 14.0 | 67.7 |  | *y*=0.005 | 8.06 | 1.3102 | 15.0 | - | 85.0 |
| *y*=0.01 | 8.83 | 1.4171 | 14.4 | - | 85.6 |  | *y*=0.01 | 9.52 | 1.5250 | 19.9 | - | 80.1 |
| *y*=0.015 | 9.50 | 1.5036 | 43.0 | - | 57.0 |  | *y*=0.015 | 8.06 | 1.2814 | 47.8 | - | 52.2 |
| *y*=0.02 | 7.16 | 1.1396 | 54.0 | - | 46.0 |  | *y*=0.02 | 9.82 | 1.5497 | 56.8 | - | 43.2 |
| *y*=0.03 | 6.77 | 1.0652 | 70.0 | - | 30.0 |  | *y*=0.03 | 6.65 | 1.0470 | 72.5 | - | 27.5 |

| *x*=0.04 | *R*_wp_ (%) | *S* | R | O | T |  | *x*=0.045 | *R*_wp_ (%) | *S* | R | O | T |
| --- | --- | --- | --- | --- | --- | --- | --- | --- | --- | --- | --- | --- |
| *y*=0 | 9.73 | 1.5730 | 18.0 | 12.0 | 70.0 |  | *y*=0 | 8.72 | 1.4113 | 16.0 | 10.0 | 74.0 |
| *y*=0.005 | 8.57 | 1.3812 | 15.5 | - | 84.5 |  | *y*=0.005 | 7.39 | 1.1942 | 15.8 | - | 84.2 |
| *y*=0.01 | 9.46 | 1.5084 | 21.0 | - | 79.0 |  | *y*=0.01 | 8.62 | 1.3716 | 27.6 | - | 72.4 |
| *y*=0.015 | 6.81 | 1.0822 | 49.6 | - | 50.4 |  | *y*=0.015 | 8.95 | 1.4210 | 50.4 | - | 49.6 |
| *y*=0.02 | 8.91 | 1.3933 | 65.5 | - | 34.5 |  | *y*=0.02 | 7.03 | 1.1134 | 67.6 | - | 32.4 |
| *y*=0.03 | 9.61 | 1.5035 | 74.0 | - | 26.0 |  | *y*=0.03 | 8.52 | 1.3377 | 75.0 | - | 25.0 |

| *x*=0.05 | *R*_wp_ (%) | *S* | R | O | T |  |
| --- | --- | --- | --- | --- | --- | --- |
| *y*=0 | 8.18 | 1.3212 | 16.3 | - | 83.7 |  |
| *y*=0.005 | 7.77 | 1.2352 | 29.2 | - | 70.8 |  |
| *y*=0.01 | 6.40 | 1.0209 | 37.4 | - | 62.6 |  |
| *y*=0.015 | 8.12 | 1.2855 | 61.3 | - | 38.7 |  |
| *y*=0.02 | 8.29 | 1.3057 | 70.0 | - | 30.0 |  |
| *y*=0.03 | 9.59 | 1.5089 | 81.3 | - | 18.7 |  |

In this work, Rietveld refinements were carried out for all the (1 – *x* – *y*)KNN-*x*BNKLZ-*y*BS ternary ceramic samples in the investigated *x* and *y* ranges. All of the XRD patterns were well refined with a high match between the fitting data and the original obsevations. Low *R*_wp_ (<10%) and *S* (<1.5) values confirmed the satisfactory selection of the phase structure mode for each composition. Figure S2 shows a typical result including the measured (solid circle) and calculated (red line) diffraction profiles together with the difference curve (pink line) for the ceramic with *x* = 0.03 and *y* = 0.01. The inset also shows the result of the amplified refinement profile in the 2*θ* range of 44 – 47 °. It is clear that the refined profile fits well to the experimental data. This ceramic has two coexisting phases of R and T. Together with the *R*_wp_ of 8.83 and the *S* of 1.4171, the phase compositions are measured to be 14.4 % in R phase and 85.6 % in T phase, showing a T-rich characteristic. The refinement results of other ceramic compositions as a function of the *x* and *y* values are given in Table S1.


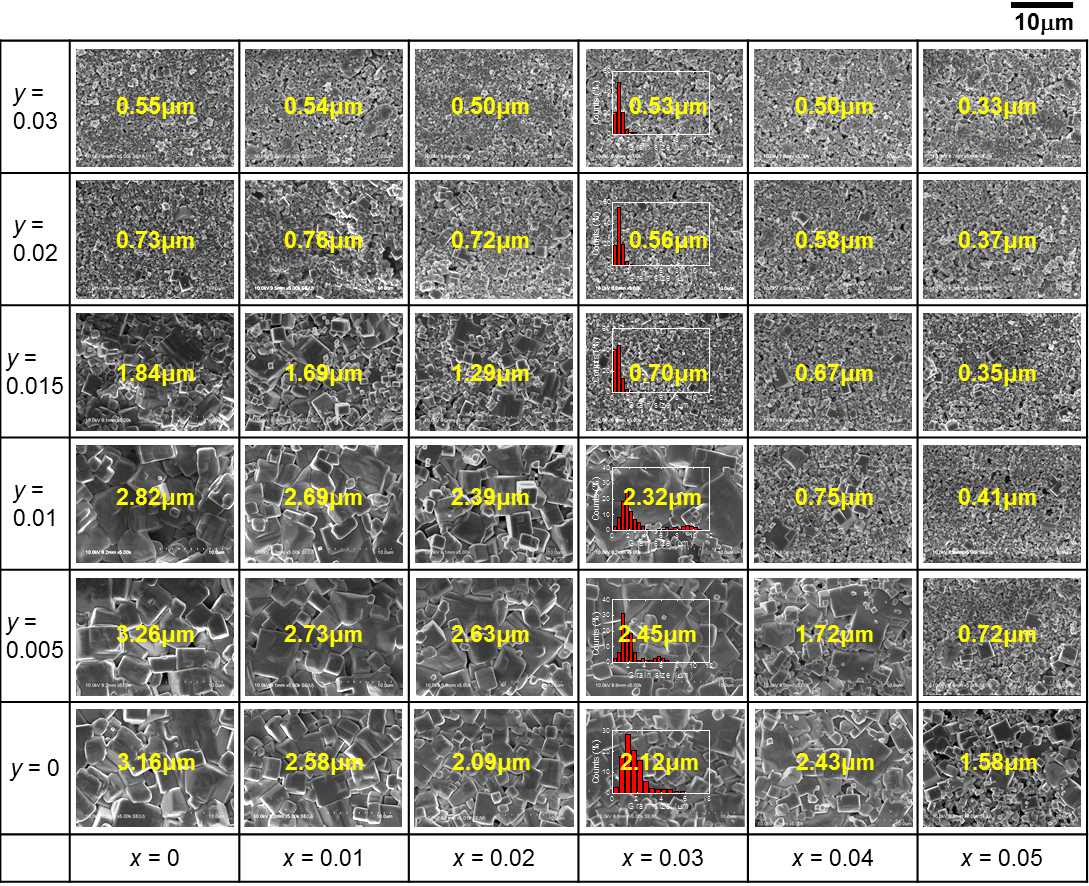


**Figure S3.** FE-SEM images of the (1 – *x* – *y*)KNN-*x*BNKLZ-*y*BS ternary ceramics (*x* = 0 – 0.05, *y* = 0 – 0.03). The values of average gain size obtained from the image analysis are presented as a function of *x* and *y* values.

Figure S3 shows the FE-SEM images of the (1 – *x* – *y*)KNN-*x*BNKLZ-*y*BS ternary ceramics as a function of both the *x* and *y* values. As the phase structure does, the grain size also greatly changes with the doping of BNKLZ and BS. Especially, the dramatic decrease in grain size can be seen when the *x* and *y* exceed certain values possibly related to the solubility limts of the dopants. A sharp decrease in grain size by excessive doping as well as an increased grain size by proper levels of doping are typical of many KNN-based ceramics doped with Bi-containing perovskites. In addition to the increased R phase content in the R-T coexistence, the decreased grain sizes are another contribution to the decline of piezoelectric activity. To investigate the effect of the grain boundary area on the piezoelectric response, the grain perimeter length substitutes for the grain size in this work (as presented in Figure 2).

**Figure S4.** Variations of the phase compositions and *d*_33_ values as a function of *x* and *y* values (redrawn from Figure 2).

Figure S4 shows the variation of *d*_33_ value and phase compositions, as the *x* changes at different *y* values, and *vice versa*. The concentration-induced peaking behavior of *d*_33_ is clear, having an intimate relationship with the fundamental roles of the R, O, T phases, as mentioned in Figure 2.

**Figure S5.** Correlation of the piezoelectric coefficient *d*_33_ with the phase composition and grain perimeter length results. a) (0.97 – *y*)KNN-0.03BNKLZ-*y*BNT ceramics (*y* = 0 – 0.04). b) (0.955 – *y*)KNN-0.45BNKLZ-*y*BG ceramics (*y* = 0 – 0.01). The results of phase composition are obtained from the Riedveld refinements.

Similar to the case for BS doping, a strong correlation between the *d*_33_ behavior and the evolution of a phase structure (i.e., constituent phase and phase composition) is also confirmed for the BNT and BG dopings (Figure S5). Notably, for the KNN-0.03BNKLZ-*y*BNT ceramic with *y* = 0.02, the enrichment of T phase up to 93.3% results in a decrease in *d*_33_ even in the R-T region (Figure S5a). This result can be a meaningful evidence that the T phase content above the optimum level adversely affect the piezoelectric performance, also supporting the existence of the optimum phase composition in a R-T phase boundary. It can also be seen from Figure S5b that even the small levels of the BG doping induces a drastic change in phase structure (R-O-T → R-T) as compared to the BNT doping. In the case of the BG doping, more precise control was thus needed to obtain the optimum R-T phase boundary structure owing to the sensitivity to formed phase structure. For instance, the optimum R-T phase boundary structure was difficult to obtain when the BNKLZ concentrations (*x*) are 0.03 and 0.04. When the *x* values are 0.03 and 0.04, the best *d*_33_ values were as low as 230.8 and 284.3 pC/N, respectively, owing to the difficulty in eliminating the O phase. However, when the *x* value increases to 0.045, we could obtain higher *d*_33_ value of 302.7 pC/N owing to the construction of near-optimum R-T phase composition via the removal of O phase.

**Figure S6.** Amplified XRD patterns (2*θ* = 44 – 47 °) measured at room temperature and the *ε*_r_–*T* curves (–150 to 200 ^o^C) measured at 100 kHz. a) (0.97 – *y*)KNN-0.03BNKLZ-*y*BNT ceramics (*y* = 0 – 0.04). b) (0.955 – *y*)KNN-0.45BNKLZ-*y*BG ceramics (*y* = 0 – 0.01).

For the KNN-0.03BNKLZ-*y*BNT (e.g., *y* = 0.015) and KNN-0.045BNKLZ-*y*BG (e.g., *y* = 0.002) systems, it should be noted that the features (i.e., peak shape and intensity ratio) of XRD peaks corresponding to the optimum R-T phase boundary structure are very similar to those of KNN-*x*BNKLZ-*y*BS system (e.g., *x* = 0.03 and *y* = 0.01). The *ε*_r_–*T* curves corresponding to the optimum R-T structure also exhibit very similar features for three KNN systems. These results indicate that the optimum R-T phase boundary structure proposed in KNN-*x*BNKLZ-*y*BS system (Figures 1 and 2) is common in doped KNN systems. In this work, it is found that the single dielectric peak forms owing to the R-T phase coexistence at near room temperature, usually followed by the peak suppression with further increasing the *x* and *y* values. It is also found from the (0.97 – *y*)KNN-0.03BNKLZ-*y*BNT ceramics that the movement of *T*_R-T_ (i.e., rhombohedral-to-tetragonal transition) below the room temperature occurs when the *y* value increases to 0.02, after the formation of the single dielectric peak at room temperature (*y* = 0.015) (Figure S6a). Finally, this results in the increased T phase content up to 93.3% in the R-T structure at room temperature, as shown in Figure S5.

**Figure S7.** The *d*_33_ vs. *E*_0_ measurement results and the variation of piezoelectric Rayleigh coefficients *d*_init_, α, and the ratio of extrinsic contribution to the total piezoelectric response, α*E*_0_/(*d*_init_+ α*E*_0_) with the phase structure and its composition. a) (0.97 – *y*)KNN-0.03BNKLZ-*y*BS ceramics (*y* = 0 – 0.03). b) (0.97 – *y*)KNN-0.03BNKLZ-*y*BNT ceramics (*y* = 0 – 0.04). c) (0.955 – *y*)KNN-0.45BNKLZ-*y*BG ceramics (*y* = 0 – 0.01).

To investigate the intrinsic and extrinsic contributions to the *d*_33_ value, Rayleigh analysis was adopted (Figures S7a – c). A series of strain-field hysteresis loops were taken with an increase in the ac-field amplitude (*E*_0_) typically smaller than 1/3~1/2 *E*_c_, at which the domain density did not change. In the Rayleigh region, the *d*_33_ value increases linearly with *E*_0_; this can be expressed by the following equation: *d*_33_(*E*_0_) = *d*_init_ + α*E*_0_. Here, *d*_init_ and α are the initial reversible piezoelectric response at a zero electric field and the piezoelectric Rayleigh coefficient, respectively. The *d*_init_ means the contribution to the piezoelectric coefficient from the lattice distortion and the reversible domain wall vibrations, and the relative magnitude of α is an excellent indicator of the extrinsic contribution originating from the irreversible motion of internal interfaces (e.g., the domain wall or phase boundaries). The change of *d*_init_ with the variation of *y* value resembles that of the small-signal *d*_33_ value. It can be seen that the values of *d*_init_, α and extrinsic contribution α*E*_0_*/(d*_init_ + α*E*_0_) are the highest at the optimum R-T phase composition (in the vicinity of 15% R and 85% T) for three KNN ternary ceramic systems (KNN-BNKLZ-BS, KNN-BNKLZ-BNT and KNN-BNKLZ- BG).

**Figure S8.** Temperature dependence of dielectric constant *ε*_r_ measured at 30 – 500 ^o^C and 100 kHz for the (1 – *x* – *y*)KNN-*x*BNKLZ-*y*BS ternary ceramics (*x* = 0 – 0.05, *y* = 0 – 0.03).

It can be seen from Figure S8 that there are different effects of the BNKLZ and BS dopings on the *T*_C_ values of the ceramics. The *T*_C_ values determined as a function of the *x* and *y* values are presented in Figure 3. In addition, one can see that there is a transition from the normal phase to diffuse ferroelectric phase (i.e., diffused phase transitions) at high doping levels of BNKLZ and BS, as usually observed in many doped KNN-based ceramics.

**Figure S9.** Properties of *T*_C_, tetragonality (c/a), *d*_33_, average grain size, grain perimeter length, phase composition, and SEM images for the ceramics possessing the near-optimum R-T phase compositions (i.e., in the vicinity of 15% in R phase and 85% in T phase).

As shown in Figure S9, the decrease in grain size can induce the decline of *d*_33_, although the ceramics possess similar phase compositions near the optimum R-T phase composition. For these ceramics, the average grain size decreases from 2.32 μm to 1.58 μm together with the correspondingly increased grain perimeter lengths, while the T phase content slightly decreases from 85.6% to 83.7%. We also note that the *T*_C_ show a nearly constant characteristic between 326 – 335 ^o^C when the ceramics have structural similarities in phase structure, phase composition and tetragonality (c/a).

**Figure S10.** The *d*_33_ vs. *T*_C_ values obtained from the polycrystalline KNN-based ceramics prepared by conventional solid-state reaction method. The values are reported from referred journals published since 2016 (Refs. 1 – 33).

Figure S10 shows the values of *d*_33_ and *T*_C_ obtained from the KNN-based ceramics prepared by conventional solid-state reaction method. It is worth mentioning that the *d*_33_ (~370 pC/N) and *T*_C_ (~332 ^o^C) values obtained in this work, as achieved by constructing the optimum R-T phase boundary condition, are almost record-high as compared to those of the recently developed polycrystalline KNN ceramics with high *T*_C_ above 300 ^o^C.

**Figure S11.** Properties of *d*_33_ and *T*_C_ values obtained from the KNN ceramics. a) (0.97 – *y*)KNN-0.03BNKLZ-*y*BS ceramics (*y* = 0 – 0.03). b) (0.97 – *y*)KNN-0.03BNKLZ-*y*BNT ceramics (*y* = 0 – 0.04). c) (0.955 – *y*)KNN-0.45BNKLZ-*y*BG ceramics (*y* = 0 – 0.01).

Figures S11a – c show both the *d*_33_ and *T*_C_ properties obtained from three different KNN ceramic systems. The dopant concentrations showing the highest *d*_33_ value induced by the construction of the optimum R-T phase boundary are different for three KNN systems. There is a clear dependency of the dopant material on the *d*_33_ value. However, it should be noted that the *T*_C_ values for the ceramics with the highest *d*_33_ values (as induced by the near-optimum R-T phase composition) are almost unchanged and maintained as high as 332 – 343 ^o^C for three KNN systems. Promisingly, these results suggest that the Bi-containing perovskites as the dopant materials can benefit the balanced development of *T*_C_ and *d*_33_ in KNN by way of achieving the optimum R-T phase boundary structure.

**References**

1. Pan, D., Guo, Y., Zhang, K., Duan, H., Chen, Y., Li, H. & Liu, H. *J. Alloys Compd.* **693**, 950–954 (2017).
2. Jiang, L., Li, Y., Xing, J., Wu, J., Chen, Q., Liu, H., Xiao, D. & Zhu, J. *Ceram. Int.*, **43**, 2100–2106 (2017).
3. Wu, W., Chen, M., Wu, B., Ding, Y. & Liu, C. *J. Alloys Compd.* **695**, 1175–1179 (2017).
4. Chen, Y., Xue, D., Chen, Z., Jiang, X., Gou, J., Liu, G., Liu, X. & Xu, Z. *Ceram. Int.* **43**, 634–640 (2017).
5. Zhao, Y., Xu, Z., Li, H., Hao, J., Du, J., Chu, R., Wei, D. & Li, G. *J. Electron. Mater.* **46**, 116–122 (2017).
6. Liu, B., Li, P., Zhang, Y., Shen, B. & Zhai, J. *J. Alloys Compd.* **695**, 2207–2214 (2017).
7. Chen, K. & Tang, J. *J. Alloys Compd.* **695**, 3364–3369 (2017).
8. Xu, K., Li, J., Lv, X., Wu, J., Zhang, X., Xiao, D. & Zhu, J. *Adv. Mater.* **28**, 8519–8523 (2016).
9. Zhang, C., Zheng, T. & Wu, J. *Ceram. Int.* **42**, 16049–16054 (2016).
10. Wang, H., Zhao, X., Xu, J., Zhai, X. & Yang, L. *Bull. Mater. Sci.* **39**, 743–747 (2016).
11. Chen, Y., Xue, D., Ma, Y., Liu, K., Chen, Z. & Jiang, X. *Phys. Lett. A* **380**, 2974–2978 (2016).
12. Zhang, K., Guo, Y., Pan, D., Duan, H., Chen, Y., Li, H. & Liu, H. *J. Alloys Compd.* **664**, 503–509 (2016).
13. Liu, B., Zhang, Y., Li, P., Shen, B. & Zhai, J. *Ceram. Int.* **42**, 13824–13829 (2016).
14. Li, Z., Zhang, Y., Li, L., Li, J. & Zhai, J. *J. Electron. Mater.* **45**, 3167–3173 (2016).
15. Jiang, L., Tan, Z., Xing, J., Wu, J., Chen, Q., Zhang, W., Xiao, D. & Zhu, J. *J. Mater. Sci.: Mater Electron.* **27**, 9812–9820 (2016).
16. Zhang, Y., Shen, B., Zhai, J. & Zeng, H. *J. Am. Ceram. Soc.* **99**, 752–755 (2016).
17. Tao, H., Wu, J. & Wang, H. *J. Alloys Compd.* **684**, 217–223 (2016).
18. Long, C., Li, T., Fan, H., Wu, Y., Zhou, L., Li, Y., Xiao, L. & Li, Y. *J. Alloys Compd.* **658**, 839–847 (2016).
19. Lv, X., Wu, J., Yang, S., Xiao, D. & Zhu, J. *ACS Appl. Mater. Interfaces* **8**, 18943−18953 (2016).
20. Guo, J., Xu, F., Shang, X., Lu, Y., Li, P., Zhou, T., Zhang, Z. & He, Y. *J. Am. Ceram. Soc.* **99**, 2341–2346 (2016).
21. Jiang, L., Xing, J., Tan, Z., Wu, J., Chen, Q., Xiao, D. & Zhu, J. *J. Mater. Sci.* **51**, 4963–4972 (2016).
22. Bucur, R. A., Badea, I., Bucur, A. I. & Novaconi, S. *J. Electron. Mater.* **45**, 3046–3052 (2016).
23. Wu, B., Wu, H., Wu, J., Xiao, D., Zhu, J. & Pennycook, S. J. *J. Am. Chem. Soc.* **138**, 15459−1546 (2016).
24. Liu, Q., Zhu, F.-Y., Zhao, L., Wang, K., Li, L. & Li, J. -F. *J. Am. Ceram. Soc.* **99**, 3670–3676 (2016).
25. Yang, Z., Zhang, X., Yang, D., Yang, B., Chao, X., Wei, L. & Yang, Z. *J. Am. Ceram. Soc.* **99**, 2055–2062 (2016).
26. Lv, X., Li, Z., Wu, J., Xi, J., Gong, M., Xiao, D. & Zhu, J. *Mater. Des.* **109**, 609–614 (2016).
27. López-Juárez, R., González-García, F. & Villafuerte-Castrejón, M. E. *J. Mater. Sci.: Mater. Electron.* **27**, 7369–7373 (2016).
28. Du, J., An, F., Xu, Z., Cheng, R., Chu, R., Yi, X., Hao, J. & Li, W. *Ceram. Int.* **42**, 1943–1949 (2016).
29. Wang, J., Luo, L., Huang, Y. & Li, W. *J. Am. Ceram. Soc.* **99**, 1625–1630 (2016).
30. Qin, Y., Zhang, J., Yao, W., Lu, C. & Zhang, S. *ACS Appl. Mater. Interfaces* **8**, 7257−7265 (2016).
31. Xiang, R. & Wu, J. *J. Alloys Compd.* **684**, 397–402 (2016).
32. Tang, X., Chen, T., Liu, Y., Zhang, J., Zhang, T., Wang, G. & Zhou, J. *J. Alloys Compd.* **672**, 277–281 (2016).
33. Zheng, T., Wu, W., Wu, J., Zhu, J. & Xiao, D. *J. Mater. Chem. C* **4**, 9779–9787 (2016).
